# Determinants of Depression among Malay Single Mothers Living in Community in Perak, Malaysia

**DOI:** 10.21315/mjms2022.29.2.8

**Published:** 2022-04-21

**Authors:** Lau YEE THENG, Razlina ABDUL RAHMAN, Shaiful Bahari ISMAIL

**Affiliations:** 1 Klinik Kesihatan Lekir, Perak, Malaysia; 2 Klinik Rawatan Keluarga, Hospital Universiti Sains Malaysia, Kelantan, Malaysia; 3 Department of Family Medicine, School of Medical Sciences, Universiti Sains Malaysia, Kelantan, Malaysia

**Keywords:** community, depression, Malaysia, prevalence, single parent

## Abstract

**Background:**

Being a single mother is challenging. Many single mothers developed depression, but some managed to survive. This study aimed to determine the proportion of depression among single mothers living in community and its associated factors.

**Methods:**

This cross-sectional study involved conveniently sampled 213 Malay single mothers living in community in Perak, Malaysia. A Malay version of Patient Health Questionnaire (PHQ)-9 was used to determine significant depression at a cut-off point of 10. Malay version of Multidimensional Scale of Perceived Social Support (MSPSS-M) and Brief-Coping with Problems Experienced (COPE) scales were used to assess support and coping strategies.

**Results:**

The proportion of depression among Malay single mothers in this study was 20.2% (95% CI: 15.0, 26.2). Previous history of depression (adjusted odds ratio [aOR]: 10.27; 95% CI: 2.18, 48.35; *P* = 0.003) and coping by using self-blame (aOR: 2.00; 95% CI: 1.45, 2.77; *P* < 0.001) increased the risk of depression. Active coping (aOR: 0.66; 95% CI: 0.46, 0.96; *P* = 0.027) and use of emotional support (aOR: 0.61; 95% CI: 0.44, 0.84; *P* = 0.002) is protective of depression.

**Conclusion:**

There was a high proportion of depression among Malay single mothers living in the community. Personal history of depression and type of coping strategies used significantly influenced the occurrence of depression among these single mothers. Providing guidance to acquire adaptive coping strategies is a crucial preventive measure.

## Introduction

Single parenthood has been associated with depression, anxiety and stress in many previous studies worldwide (1–2). With the increasing trend of families headed by single mothers (3), it is worrying that the prevalence of depression is also on the rise. In Malaysia, there were 161,227 single mothers registered with the Office of Women Development in 2021 (4). The most common causes that lead to single motherhood in this country are divorce, separation and widowhood (5). In Perak, the number of single mothers has jumped from 5,442 in 2017 (6) to 10,977 in 2021 (4), with 63.5% accounting for Malay single mothers, while 11.6% and 18.9% were Chinese and Indian, respectively. This significant increase mirrored the increase of 12% in the number of divorces that occurred among the ethnic groups (7).

Single mothers are at a higher risk of depression as compared to partnered mothers (1, 8–10). This view was consistent with the findings from the National Health and Morbidity Survey (NHMS) 2019, which showed that prevalence of depression among the divorced or widowed women was higher (2%) compared to those who are married (1.8%) in the country (11). Single mothers are considered as among the most economically and socially affected group. The consequences of being single mothers, such as adversities in life, poverty, lack of social support and social exclusion, were the main aspects that predisposed them to depression (8). This was also reflected in the NHMS report, which showed that higher prevalence of depression was associated with female gender, single, bottom 40% income and those of Malay ethnicity (11). Undeniably, single mothers are recognised to suffer from higher level of stress due to their multitasking duties as they need to balance between work and household duties. They also experience child-related stress as they need to shoulder multiple responsibilities, such as raising up children, finding financial resources, and managing household (12). In addition, they must also cope with emotional stress and stigma of their status. Prolonged exposure to these stressors leads to frustration, helplessness and fatigue, which may result in depression.

Nonetheless, not all single mothers developed depression. This is because some of them coped very well with their predicament. This finding indicates that coping strategy plays a very important role in determining the risk of depression. Coping strategies, such as being optimistic, proper planning and use of emotional support can prevent single mothers from getting depression, while non-productive coping, such as criticising, will increase the level of stress among single mothers (13). Therefore, it is necessary to determine how single mothers in Malaysia cope with their stressors and the type of coping strategies they adopt to enable the policy makers and stakeholders to design preventive interventions for this unfortunate marginalised population.

Many of previous studies on similar population were conducted on young single mothers living in institutions due to premarital pregnancies. However, the majority of them are living in the community, and thus, the findings of previous studies may not really represent the real picture. Hence, there is a need for a community study to determine the prevalence of depression among them, if any. Early detection of depression among single mothers living in the community is crucial to help provide prompt intervention in a timely manner to improve their psychological wellbeing in the later part of their life. Thus, the aims of this study were to determine the proportion of depression among Malay single mothers living in the community and to identify the underlying factors.

## Methods

Most single mothers live in the community and many of them survive with support from non-governmental organisations (NGO). Due to their scattered location, it is not feasible to locate the single mothers individually. Thus, for the purpose of this cross-sectional study, members of Persatuan Ibu Tunggal Nur Kasih were chosen to represent the single mothers living in the community. This NGO was chosen since it has the largest number of members (about 250 members) compared to others and all its members were living independently in the society. Inclusion criteria were NGO members with age 18 years old and above, who understood Bahasa Malaysia, and had consented to the study. Members who were illiterate or absent during data collection day were excluded. The optimum sample size was calculated using single proportion formula with precision of 0.07 with 95% confidence level and prevalence of depression among single mothers is 60.5% (14). Sample size for factors associated with depression among single mothers was calculated using the formula for comparing two proportions for categorical variables and comparing two means for numerical variables using PS software. The largest sample size resulted from calculation for prevalence and, after inclusion of 10% dropout rate, the final estimated sample size was 206.

### Research Tools

The research tools used in this study comprised of three previously validated Malay version questionnaires in addition to the sociodemographic data of the participants. The Patient Health Questionnaire-9 (PHQ-9) was modified from the depression module of PHQ and consists of nine statements based on the diagnostic and statistical manual of mental disorders (DSM)-IV criteria for major depression (15). The items were scored from 0 (not at all) to 3 (nearly every day) and the severity of depression may be assessed based on the scores that ranged from 0 to 27 (15). PHQ-9 has been shown to be accurate in diagnosing major depression (16) and had been translated into more than 30 languages. The Malay version of PHQ-9 has been validated among women who attended primary care clinic and has good internal reliability (Cronbach’s alpha = 0.70) with a sensitivity of 87% (95% CI: 71, 95) and a specificity of 82% (95% CI: 74, 88) (17). Scores of 5–9 predominantly represent patients with either no depression or at subthreshold of depression, and thus, in this study, scores of 10 and above were considered as having depression (15, 17).

The second questionnaire was Malay version of Multidimensional Scale of Perceived Social Support (MSPSS-M). This instrument has been tested in different population sets and has shown good internal consistency (Cronbach’s alpha = 0.89–0.93) and test-retest reliability (0.77–0.89) (18–20). The negative correlation of the scores of the instrument with the Malay version of Medical Outcomes Study, Including Social Support Survey (MOS-SSS), General Health Questionnaire (GHQ-12), Beck Depression Inventory (BDI) and Geriatric Depression Scale (GDS), confirmed its validity (18, 20). MSPSS-M has 12 items and is used to assess social support from three sources: family, friends, and significant others. Items 1, 2, 5 and 10 assessed support from significant other; items 3, 4, 8 and 11 assessed family support; and items 6, 7, 9 and 12 assessed friends’ support. Each item has a seven-point Likert-scale ranging from ‘very strongly disagree’ (1 point) to ‘very strongly agree’ (7 points). The degree of social support was determined by the sum of the points for the items. The greater the total score, the higher the social support. For each subscale, the respective items need to be summed, and then, dividing by 4. The total score was determined by adding the points for all 12 items, and then, dividing by 12. Mean total score ranging from 1 to 2.9 was considered low support, a score of 3–5 was considered moderate support and a score from 5.1–7 was considered high support (21).

The third questionnaire was Malay version of Brief-Coping with Problems Experienced (COPE) scale, which made up of 14 subscales, including self-distraction, active coping, denial, substance use, use of emotional support, use of instrumental support, behavioural disengagement, venting, positive reframing, planning, humour, acceptance, religion and self-blame (22). The scale comprises of 28 items (two items per scale) and is rated by a four-point Likert scale ranging from ‘I haven’t been doing this at all’ (1 point) to ‘I have been doing this a lot’ (4 points). Scores are the sum of appropriate items for each scale and ranged from 2 (minimum) to 8 (maximum). There is no reverse scoring and higher scores indicate greater use of that specific coping strategy among respondents (22). The Malay version of Brief-COPE has previously been validated among cancer patients and medical students and showed variable internal consistencies ranging from 0.51 to 0.99 (23) and an overall Cronbach alpha of 0.83 (24). The factor analysis of the items showed that construct validity of the instrument was comparable to the original Brief-COPE (24).

### Data Collection

The best way to gather single mothers living independently in the community is through the NGO that caters to their needs. Persatuan Ibu Tunggal Nur Kasih in Ipoh, Perak, caters to single mothers from all over the state of Perak and thus was a suitable choice for this cross-sectional study. With the collaboration of the NGO, a session was planned to gather the members for data collection. The members who attended the session were briefed regarding the study and the eligibility criteria. Attendees who fulfilled the eligibility criteria were invited to participate in the study. The study employed convenient sampling since it was deemed the most appropriate to obtain the maximum number of samples within the study duration. All members were ensured that the gathered information will be kept confidential. Their participation should be voluntary and, in case they decided not to participate, it would not have any bearing on the privilege they received from the NGO. Further briefing and instruction on questionnaires were given to those who agreed to participate. Following submission of their informed consent form, a set of self-administered questionnaires was distributed to the each of the participants. Each participant was given about 30 minutes to complete the questionnaires. Participants were not allowed to discuss while answering the questionnaire to avoid contamination of the data. The NGO staff was not allowed to be present during data collection to avoid bias. The researchers stayed close the participants to help if there were any questions or inquiries. At the end of 30 min, the researchers collected the questionnaires from the participants and checked for possible missing data. In the interest of confidentiality, all forms in this study were anonymous. Only research team members could access the data that was stored in a password-protected computer and could only be accessed by researchers and their team members. All participations were voluntary and any dissent on their part did not interfere with their privileges from the association. Participants who were detected to have high scores, which indicated depression, were referred to the nearest health clinic for further evaluation and management.

### Data Analysis

The data were analysed using the SPSS version 24 (25). Descriptive analysis was done where numerical variables were expressed as mean and standard deviation, while categorical variables were expressed as frequency and percentage. Categories with small numbers were identified and meaningful combinations of categories were built. Simple and multiple logistic regressions were carried out to determine the factors associated with depression among Malay single mothers, where the dependent variable was the depression status. Simple logistic regression was used to screen for potential depression-associated factors. Variables with *P*-value less than 0.25 and clinically significant variables were used for the multiple logistic regression to further confirm the depression-associated factors. Backward and forward stepwise procedures were used for variable selection until all important variables were included in the model. All possible two-way interactions and multicollinearity were checked before preliminary model was finalised. Fitness of the model was tested by Hosmer and Lemeshow goodness of fit test, the classification table and receiver operating characteristics (ROC) curve.

## Results

A total of 213 single mothers agreed to participate and all had successfully returned the questionnaires, giving a response rate of 100%. The age of the respondents ranged from 25 years old to 84 years old with a mean of 63 years old (SD 11.6) while the median duration for being a single mother was 10 years (interquartile range [IQR]: 11). Most of them belonged to bottom 40% socioeconomic group with median income of RM700 (IQR: 655). More than half of the participants had no dependents, while some had up to seven dependents. Majority of the participants who were not depressed perceived that they had high social support from family, friends, and significant others, while those in depressed group perceived they had moderate support from all categories. The detailed sociodemographic and clinical data between the groups are summarised in Table 1.

In terms of coping strategies, regardless of depressed status, the most common strategy used was religion while, the least common strategy was substance abuse (Table 2). The proportion of depression among Malay single mothers in this study was 20.2% (95% CI: 15.0, 26.2). Table 3 lists the variables that showed significant association with depression after simple logistic regression. Previous history of depression, active coping, use of emotional support, and self-blame were significantly associated with depression (Table 4). Those with a history of depression were 10 times more likely to develop depression (95% CI: 2.18, 48.35; *P* = 0.003), while those using self-blame for coping exhibited twice the odds of developing depression (95% CI: 1.45, 2.77; *P* < 0.001). On the other hand, those using active coping and emotional support exhibited 34% (95% CI: 0.46, 0.96; *P* = 0.027) and 39% (95% CI: 0.44, 0.84; *P* = 0.002) lesser odds of developing depression, respectively.

## Discussion

Several previous studies have reported that single mothers exhibit a higher prevalence of depression (33%–38.1%) compared to general population (10, 26–28). The current study reported that the proportion of depression among Malay single mothers living in community and attending a non-governmental organisation in Perak was 20.2%. However, this value was low compared to that reported in a previous local community study, which found that about 60.5% of single mothers were depressed (14). The lower prevalence of depression in our study may be attributed to the facts that Abdul Kadir and Bifulco (14) had bigger sample size, and thus, able to detect a higher number of single mothers who were depressed. In addition, the fact that they were members of an organisation that provides support to these individuals also helped. Among the various programs provided by the NGO are cooking and sewing class, health information talks and life management skills, such as financial management talk. All these aimed to empower single mothers and equipped them with better entrepreneur skills through education (29). In addition, many of them reported very high support from friends, families, and significant others. These supports potentially help them to accommodate to new and unexpected experiences and alleviate various problems they faced.

Secondly, many of our respondents were older and had no dependents and thus have less economic burden. In the study conducted by Abdul Kadir and Bifulco (14), majority of the single mothers (62.7%) had children at home who were under 18 years old, and thus, these mothers would be under more stressful situation compared to our cohort. The more the dependents, the higher the burden one needs to cater. Higher burden will lead to greater economic deprivation, which will then result in higher risk of depression among single mothers (26). Furthermore, the lower number of those having depression may also be attributed to the religion of participants. Muslim Malay mothers were less likely to yield to feelings of depression as local customs and religiosity encouraged them to be more accepting (*redha*) to what had happened to them in accordance with the Islamic teachings that encourage its believers to have positive thoughts and to become more resilient and tolerant towards adverse life events (30).

Despite this, those with history of depression have difficulties to escape depression since about 40%–60% of people who suffered a previous episode of depression will have increased risk of recurrence (31). In the current study, we found that those with previous history of depression have a 10-time higher risk of depression compared to other single mothers. This finding indicates that single mothers with history of depression are highly vulnerable to develop further depressive episodes and need to be cared for. This finding is not unexpected since being a single mother, these women may be scorned by society, coupled with loss of emotional and financial support, and if they have children that they need to care for, the burden that they must carry are enormous, thus making those at risk of depression develop a full-blown episode. Nevertheless, some of them may still survive if they have good social support. In this study, majority of the participants perceived they had very good support from all quarters, including family, friends and significant others. Thus, all the perceived social support showed negative association with depression in Malay single mothers. Furthermore, the participants were active members of the NGO, and thus, they were not isolated and were provided with very good support.

In addition to support, one’s inner strength in the form of coping strategies is important to ones’ physical and psychological health, as stress will lead to poor psychological outcomes (32). Based on differing social values, different individuals will utilise different coping strategies to deal with stressors in life. In this community-based study, we found that coping strategies hold the greatest influence on the risk of depression among single mothers apart from having previous history of depression. One of the most frequently used coping strategies among the Malay single mothers in this study was religion. Religion plays a vital role as a resource for people to cope with difficulties and is commonly used as a buffer for stress and depressive symptoms (33–35). Since the studied participants were Malay Muslim, it is not surprising that the Islamic beliefs, which encourages people to be optimistic, consider God’s mercy and have endurance in dealing with adverse events in life, had a high influence as a coping mechanism adopted by the participants. Whenever there were challenges in life, they sought comfort by prayers and recitation of their Holy Book as strategies to cope with stressors in life (36).

Among the significant coping strategies that protected the participants against depression were active coping and use of emotional support. Those who used active coping exhibited flexibility to unexpected circumstances, perceived stress as challenges, and actively coped with difficulties in life. This coping mechanism allowed the single mothers to face their challenges in a positive way. The greater the use of active coping, the less the likelihood of having depression, as it can protect against the adverse effects of stress. Similar coping strategies were adopted by university and nursing students to avoid depression (37–38). It is noteworthy that the mean age for our study cohort was 63 years old and the mean duration of being a single mother was around 10 years. It is possible that the matured age when they became single mothers and their long-term status as single mothers had influenced them to adopt active coping as their strategy to survive.

Coping through use of emotional support was also protective against depression. This adaptive emotion-focused coping strategy plays a crucial role in managing long-term events, similar to when coping with chronic or terminal illnesses (39). For example, women with cancers who used emotional support as coping strategy exhibited lower depression score and better quality of life (40–41). In the case of single mothers, they obtained emotional support from family members or friends as well as existing social support systems, such as governmental agencies, welfare institutions, or experts. In this study, the single mothers received not only social but also emotional support through their involvement in the organisation. Socially, the NGO provided networking platform for them to build social skills and help them resolve troubling life events. The emotional support provided by social ties creates positive reinforcement and enhances mental health as the mothers were able to cope with their hardships. In addition, the frequent meetings generated a sense of belonging where they can share common feelings and empathies with each other (29).

In this study, the negative coping strategies were less utilised compared to the positive ones. Nonetheless, we found that use of self-blame as a coping strategy was associated with increased level of depression. This finding was parallel to those of previous studies, which showed that avoidant coping strategy, particularly self-blame, was associated with depression (40, 42–47). Self-blame is usually related to one’s unrealistic or false belief that one must be perfect in doing things. This negative coping can cause one to doubt their abilities, tend to amplify perceived inadequacies in dealing with stressors, and hence, lead to self-criticism (45). If this occurs for longer duration, it may have tremendous implications over ones’ emotional and mental health, leading to depression.

## Conclusion

Single mothers are at higher risk of depression due to the many challenges they need to face. There is a high proportion of depression among Malay single mothers living in community in Perak compared to what was found in general Malaysian population. Having had history of depression and practicing self-blame increases the risk of developing depression in these single mothers. On the other hand, the use of active coping and emotional support as coping strategies significantly protected them against depression. These findings highlighted that intervention is crucial at community level to support this vulnerable population. Preventive intervention targeted on acquiring adaptive coping strategies is crucial to be introduced among single mothers to enhance their mental health and to prevent maladaptive coping strategies.

To the best of our knowledge, this is the first study that included determination of risk of depression among single mothers living in community. However, the results of this study are only applicable to Malay single mothers and may not be extrapolated to other ethnic groups in Malaysia. Hence, future studies involving single mothers from other ethnic groups are needed so that the findings can be extrapolated to all single mothers throughout Malaysia. A longitudinal study should be done in future to strengthen the findings of this study and provide a detailed picture of the mental health of single mothers.

## Figures and Tables

**Table 1:** Socio-demographic and other characteristics of depressed and non-depressed Malay single mothers in Perak, Malaysia (*n* = 213)

Variables	Not depressed (*n* = 170)		Depressed (*n* = 43)	

	mean (SD)	*n* (%)	mean (SD)	*n* (%)
Age (years old)	62.2 (11.57)		64.2 (11.59)	
Educational level				
Primary or less		69 (40.6)		21 (48.8)
Secondary and higher		101 (59.5)		22 (51.2)
Employment				
Unemployed		98 (57.6)		27 (62.8)
Employed		72 (42.4)		16 (37.2)
Duration of being single mother				
Less than 10 years		84 (49.4)		17 (39.5)
10 years or more		86 (50.6)		26 (60.5)
Number of dependents				
No dependents		91 (53.5)		25 (58.1)
1–2 dependents		56 (32.9)		6 (14.0)
3 or more dependents		23 (13.5)		12 (27.9)
Monthly household income				
Less than RM1,000		99 (58.2)		30 (69.8)
RM1,000 or more		71 (41.8)		13 (30.2)
Family history of depression				
No		169 (99.4)		39 (90.7)
Yes		1 (0.6)		4 (9.3)
Underlying chronic medical illness				
No		71 (41.8)		25 (58.1)
Yes		99 (58.2)		18 (41.9)
Previous history of depression				
No		167 (98.2)		36 (83.7)
Yes		3 (1.8)		7 (16.3)
Family support				
High		132 (77.6)		16 (37.2)
Moderate		37 (21.8)		25 (58.1)
Low		1 (0.6)		2 (4.7)
Friends’ support				
High		117 (68.8)		15 (34.9)
Moderate		52 (30.6)		26 (60.5)
Low		1 (0.6)		2 (4.7)
Significant others’ support				
High		112 (65.9)2		13 (30.2)
Moderate		57 (33.5)		7 (62.8)
Low		1 (0.6)		3 (7.0)

**Table 2:** Mean coping strategy score and comparison of coping strategy used between not depressed and depressed Malay single mothers living in the community in Perak, Malaysia (*N* = 213)

Coping strategy	Scores	Not depressed (*n* = 170)	Depressed (*n* = 43)	*P*-value

	mean (SD)	a*n* (%)	a*n* (%)	
Religion	7.55 (0.97)	152 (71.4)	33 (15.5)	0.028
Active coping	6.78 (1.37)	125 (58.7)	8 (3.8)	<0.001
Use of instrumental support	6.57 (1.12)	122 (57.3)	14 (6.6)	<0.001
Acceptance	6.50 (1.09)	110 (51.6)	16 (7.5)	0.001
Use of emotional support	6.33 (1.72)	111 (52.1)	4 (1.9)	<0.001
Positive reframing	6.33 (1.15)	82 (38.5)	9 (4.2)	0.001
Planning	6.31 (1.21)	100 (46.9)	15 (7.0)	0.005
Self-distraction	6.16 (1.03)	55 (25.8)	16 (7.5)	0.546
Humour	5.70 (1.09)	28 (13.1)	9 (4.2)	0.490
Venting	5.39 (1.26)	32 (15.0)	9 (4.2)	0.754
Denial	5.12 (1.50)	26 (12.2)	5 (2.3)	0.542
Self-blame	3.60 (1.32)	0 (0)	1(0.5)	0.046
Behavioural disengagement	3.55 (1.64)	15 (7.0)	2 (0.9)	0.367
Substance use	2.05 (0.29)	*	*	

Notes:

a Refer to those who scores high (7–8) for the type of coping strategy;

* None of the participants score high in this type of coping strategy

**Table 3:** Factors associated with depression among Malay single mothers by simple logistic regression

Variables	β	Crude OR (95%)	Wald stat (df)	*P*-value
Age (years old)	0.015	1.02 (0.99, 1.05)	0.997	0.318
Educational level				
Primary or less	0	1		
Secondary and higher	−0.334	0.72 (0.37, 1.40)	0.952	0.329
Employment				
Unemployed	0	1		
Employed	−0.215	0.81 (0.41, 1.61)	0.374	0.541
Duration of being single mother				
Less than 10 years	0	1		
10 years or more	0.401	1.49 (0.76, 2.95)	1.333	**0.248**
No of dependents				
No dependents	0	1		
1–2 dependents	−0.942	0.39 (0.15, 1.01)	3.765	**0.052**
3 or more dependents	0.641	1.90 (0.83, 4.34)	2.314	**0.128**
Monthly household income				
Less than RM1,000	0	1		
RM1,000 or more	−0.504	0.61 (0.29, 1.24)	1.888	**0.169**
Family history of depression				
No	0	1		
Yes	2.853	17.33 (1.89, 159.40)	6.350	**0.012**
Underlying chronic medical illness				
No	0	1		
Yes	−0.661	0.52 (0.26, 1.02)	3.648	**0.056**
Previous history of depression				
No	0	1		
Yes	2.382	10.82 (2.67, 43.88)	11.124	**0.001**
Family support				
High	0	1		
Moderate	2.803	16.50 (1.42, 192.34)	5.005	**0.025**
Low	1.718	5.57 (2.70, 11.52)	21.532	**<0.001**
Friends’ support				
High	0	1		
Moderate	2.747	15.60 (1.33, 182.58)	4.791	**0.029**
Low	1.361	3.90 (1.91, 7.97)	13.937	**<0.001**
Significant others support				
High	0	1		
Moderate	3.252	25.85 (2.50, 266.95)	7.453	**0.006**
Low	1.406	4.08 (1.96, 8.51)	14.083	**<0.001**
Coping Strategy				
Self-distraction	0.115	1.12 (0.81, 1.56)	0.468	0.494
Active coping	−0.700	0.50 (0.38, 0.65)	27.706	**< 0.001**
Denial	−0.092	0.91 (0.73, 1.14)	0.679	0.410
Substance use	−0.475	0.62 (0.13, 3.03)	0.345	0.557
Use of emotional support	−0.601	0.55 (0.44, 0.68)	29.847	**< 0.001**
Use of instrumental support	−0.615	0.54 (0.40, 0.73)	16.223	**< 0.001**
Behavioural disengagement	0.047	1.05 (0.86, 1.28)	0.209	0.647
Venting	−0.051	0.95 (0.73, 1.24)	0.140	0.708
Positive reframing	−0.510	0.60 (0.44, 0.81)	10.832	**0.001**
Planning	−0.435	0.65 (0.49, 0.85)	10.002	**0.002**
Humour	−0.099	0.91(0.67, 1.23)	0.408	0.523
Acceptance	−0.535	0.59 (0.43, 0.79)	12.246	**< 0.001**
Religion	−0.528	0.59 (0.43, 0.81)	10.392	**0.001**
Self-blame	0.544	1.72 (1.31, 2.27)	14.906	**< 0.001**

Note: All the bold numbers were included in the multiple logistic regression

**Table 4:** Factors associated with depression among Malay single mothers by multiple logistic regression

Variables	Simple logistic regression				Multiple logistic regression			

	β	Crude OR (95%)	Wald stat (df)	*P*-value	Adjusted β	Adjusted OR a(95%)	Wald stat (df)	*P*-value
Previous history of depression								
No	0	1			0	1		
Yes	2.382	10.82 (2.67, 43.88)	11.124	**0.001**	2.329	10.27 (2.18,48.35)	8.686	**0.003**
Coping Strategy								
Active coping	−0.700	0.50 (0.38, 0.65)	27.706	**< 0.001**	−0.411	0.66 (0.46, 0.96)	4.884	**0.027**
Use of emotional support	−0.601	0.55 (0.44, 0.68)	29.847	**< 0.001**	−0.495	0.61 (0.44, 0.84)	9.256	**0.002**
Self-blame	0.544	1.72 (1.31, 2.27)	14.906	**< 0.001**	0.694	2.00 (1.45, 2.77)	17.576	**< 0.001**

Notes:

a Adjusted β is adjusted Beta coefficient; Backward & Forward LR Multiple logistic regression model was applied; Hosmer-Lemeshow test not significant (*P* = 0.197); Overall classification table = 85.4%; Area under the ROC curve = 88.2%; The model fitted well. Model assumptions were met. There was no multicollinearity nor interaction found
